# A New Perspective to Tribocharging: Could Tribocharging Lead to the Development of a Non-Destructive Approach for Process Monitoring and Quality Control of Powders?

**DOI:** 10.3390/foods11050693

**Published:** 2022-02-26

**Authors:** Hadi Mehrtash, Dinara Konakbayeva, Solmaz Tabtabaei, Seshasai Srinivasan, Amin Reza Rajabzadeh

**Affiliations:** 1 Department of Mechanical Engineering, McMaster University, Hamilton, ON L8S 3L8, Canada; mehrtash@mcmaster.ca; 2 Department of Chemical Engineering, Howard University, Washington, DC 20060, USA; dinara.konakbayeva@nu.edu.kz; 3 W Booth School of Engineering Practice and Technology, McMaster University, Hamilton, ON L8S 0A3, Canada

**Keywords:** tribocharging, rapid characterization, computational fluid dynamics (CFD), artificial neural network (ANN)

## Abstract

This study explores a new perspective on triboelectrification that could potentially lead to the development of a non-destructive approach for the rapid characterization of powders. Sieved yellow pea powders at various particle sizes and protein contents were used as a model system for the experimental charge measurements of the triboelectrified powders. A tribocharging model based on the prominent condenser model was combined with a Eulerian–Lagrangian computational fluid dynamics (CFD) model to simulate particle tribocharging in particle-laden flows. Further, an artificial neural network model was developed to predict particle–wall collision numbers based on a database obtained through CFD simulations. The tribocharging and CFD models were coupled with the experimental tribocharging data to estimate the contact potential difference of powders, which is a function of contact surfaces’ work functions and depends on the chemical composition of powders. The experimentally measured charge-to-mass ratios were linearly related to the calculated contact potential differences for samples with different protein contents, indicating a potential approach for the chemical characterization of powders.

## 1. Introduction

Powders and their homogeneous blending play vital roles in the food, agrochemical, pharmaceutical, and other manufacturing industries [1,2,3,4,5]. Most of the food products in the market are powder-based (e.g., milk powders, protein powders, flours), and the development of such products with acceptable quality requires an understanding of powder characteristics and performance in terms of flowability, processability, and handling. For this, multiscale knowledge is required for understanding the influence of different factors on the powder performance, i.e., material, process, and environmental properties. As a result, the powder performance could be controlled and engineered to ensure optimal properties of the final products. Powders are often characterized based on their chemical (e.g., protein, carbohydrate, and mineral content) and physical properties (e.g., particle size, density, flowability, and dispersibility), which affect their manufacturing process and the quality of the final products. Current characterization methods for the quality control of powders are time-consuming, expensive, and require a highly trained workforce. Furthermore, most of them are after-the-fact methods that cannot prevent the enormous economic loss due to the high rate of sub-standard production before identification of the processing problem [6]. Traditional wet chemistry methods, such as Kjeldahl and enzyme digestion approaches, for determining the protein and carbohydrate contents of powders are time-consuming and require large amounts of chemicals. Recently, the feasibility of spectroscopic methods for the offline characterization of organic powders has been addressed [7]. In the pharmaceutical industry, real-time process monitoring is essential to ensuring consistent dosage of final products [8]. During milk powder processing, a rapid analytical method is needed for quality control to avoid final product rejection [9]. Therefore, non-destructive process monitoring and quality control of powders based on their physicochemical properties is a critical competitive advantage in the powder industry.

Triboelectrification of powders is caused by interparticle collisions and particle–wall interactions during powder handling, grinding, sieving, blending, pouring, and conveying in particle-laden flows. Tribocharging is the phenomenon of electric charge transfer between different materials after any type of contact, such as rolling, sliding, and impact [1]. From an industrial perspective, triboelectric charging has drawn substantial attention over the years due to its hazardous effects. The triboelectrification of powders is often considered a nuisance phenomenon, especially during the powder handling process in various industries. In particular, in the pharmaceutical industry, wall-fouling occurs due to the electrostatic charging of particles during transport in particle-laden pipe flows, leading to inconsistencies in final product dosage [10]. Dust explosions due to excessive powder charging also pose a severe hazard that can cause damages to infrastructure and equipment, and harm personnel [11]. Moreover, in the polyolefin industry, particle charging combined with extremely exothermic polymerization reactions leads to the formation of large sheets of melted particles, which lowers the performance of the reactor, resulting in reactor shutdown [12].

The electrostatic separation of agro-materials is another promising application of triboelectrification of particles at laboratory and industrial scales. Several researchers have investigated the application of triboelectric separation for the dry fractionation of plant-based materials to obtain fiber-, starch-, and protein-enriched fractions [13,14,15,16,17,18,19,20]. In the recycling industry, tribocharging has been used for the electrostatic separation of plastics [21]. The application of electrostatic separation for the dry beneficiation of coal has also been addressed [22]. 

Electrostatic charge generation and triboelectrification in particle-laden pipe flows have been broadly studied in the literature. However, little attention has been paid to utilizing this natural phenomenon as a tool for the characterization of powders’ physical and chemical properties, which is the focus of this study. The modeling of particle tribocharging, based on the physicochemical properties of particles and the hydrodynamics of the system, would provide insight into the charging behavior of powders, supporting the development of a predictive tool for the rapid and cost-effective characterization of powders.

## 2. Background Theory

Two different types of contacts may occur during particle transport in particle-laden flows: particle-particle interaction and particle–wall interaction [23]. In the dilute phase, inter-particle collisions are negligible. As a result, the amount of charge transfer via particle–particle collisions is insignificant. Thus, the primary mechanism of charge transfer in dilute particle-laden flows is particle–wall interactions. The calculation of transferred charge between particles and the wall could be described based on the condenser model, which considers particle and wall surfaces as the two plates of a capacitor where charge transfer occurs due to different work functions of two materials in contact [24,25,26,27,28,29,30]. The condenser model describes the electron transfer process between contact surfaces in terms of a capacitor. The contact surfaces are considered as the plates of a capacitor in which charge transfer occurs due to the potential difference between the two plates [27]. In particle-laden pipe flows, particles repeatedly collide with the inner wall, due to which tribo-charging takes place. Based on the condenser model, it is possible to formulate the particle charge generated by repeated impacts in a gas-solids pipe flow. In this study, a tribocharging model was employed based on repeated particle impacts on the wall, as proposed by Matsusaka and Masuda [31]. According to this model, when a particle moves from point *x* to point *x +* ∆*x* along the pipe axis, the variation of charge per mass ratio of particles as a function of the number of particle–wall collisions (*n*) is derived from the following exponential equation:

(1) $$\begin{array}{l} \frac{∆q}{m_{p}}=q_{m}\left(x+∆x\right)-q_{m}\left(x\right)\\ =\left(q_{m\infty }-q_{m0}\right)\left\{exp\left(-\frac{n\left(∆x\right)}{n_{0}}\right)\right\}\;\left\{1-exp\left(-\frac{n\left(∆x\right)}{n_{0}}\right)\right\}, \end{array}$$

where $q_{m0}$ and $q_{m\infty }$ are the particle charge per mass ratios at *x =* 0 and *x =* ∞, respectively, and $n_{0}$ is the dimensionless characteristic number of particle electrification. Assuming that the initial charge per mass of particles is negligible ($q_{m0}\approx 0$), the difference between the initial and final charge of particles is equal to total transferred charge per mass and is calculated by

(2) $$\frac{∆q}{m_{p}}=\frac{6\varepsilon_{0}V_{c}n\left(∆x\right)kS}{\pi D_{p}^{3}\rho_{p}z_{0}}.$$

where *ε*_0_ is the absolute permittivity of gas (8.854 × 10^−12^ F m^−1^), *k* is dimensionless charging efficiency, and *z*_0_ is the critical gap between the particle and the wall. It must be noted that the value of transferred charge per mass depends on both physical properties and the material of contacting surfaces. Collision number (*n*), contact area (*S*), pipe length (∆*x*), particle size (*D_p_*), and density ($\rho_{p}$) are physical properties that influence the tribocharging of particles. On the other hand, contact potential difference ($V_{c}$) is an essential factor that depends on the work functions and is determined by the chemical composition of the contacting surfaces. Assuming that particles are spherical and elastic with a smooth surface, the maximum contact area during impact with a wall can be calculated based on the equation proposed by Matsusaka and Masuda [31],

(3) $$S=1.36k_{e}^{2/5}\rho_{p}^{2/5}D_{p}^{2}v_{i}^{4/5},$$

where *k_e_* is the elasticity parameter, *ρ_p_* is the particle density, *D_p_* is the particle diameter, and *v_i_* is the impact velocity of the particle. The elasticity parameter is calculated based on the following equation:

(4) $$k_{e}=\frac{1-v_{1}^{2}}{E_{1}}+\frac{1-v_{2}^{2}}{E_{2}}$$

where *E* is the Young’s modulus, *v* is the Poisson’s ratio, and subscripts 1 and 2 indicate the particle and the wall, respectively.

## 3. Materials and Methods

### 3.1. Materials

Pin-milled yellow pea flour (*Pisum sativum*) containing 19.8 wt% protein (wet-basis) was purchased from Canadian International Grains Institute (CIGI, Winnipeg, MB, Canada). The flour was stored at −20 °C. Potassium sulfate crystallized (99.9%) was purchased from VWR (Radnor, PA, USA), and selenium dichloride oxide (99%) was provided from Alfa Aesar (Haverhill, MA, USA). Nessler reagent was obtained from Ricca Chemical Company (Arlington, TX, USA) and was used for the Kjeldahl analysis to determine protein contents.

### 3.2. Sieving

The yellow pea flour was dried for 48 h in an oven at 103 °C prior to the separations. The flour was manually separated by passing it through six 8-inch stainless steel wire mesh sieves with the following U.S. Standard Sieve numbers: 60-mesh (250-μm), 80-mesh (177-μm), 100-mesh (147-μm), 140-mesh (106-μm), 200-mesh (75-μm), and 270-mesh (53-μm). These sieves with different aperture sizes were picked in order to obtain several fractions of different particle sizes and compositions. As a result, six different size fractions retained on the sieves plus one fraction from the bottom collecting pan. All yellow pea sieved fractions were analyzed for their protein content, particle size, and tribo-charging behavior. 

### 3.3. Analytical Methods

The protein content of sieved fractions was determined using a chemical protein digestion method (N × 6.25, Kjeldahl analysis) [29] to investigate the influence of the chemical composition of powders on the charging behavior of particles. The volume-fraction size distribution of all sieved fractions were determined by Mastersizer 2000 (model APA 2000, Malvern Instruments Ltd., Malvern, UK) with a dry dispersion unit of Scirocco 2000 (model ADA 2000, Malvern Instruments Ltd., Malvern, UK) and used to calculate the particle volume mean diameters (D4,3).

### 3.4. Charge Measurements

**Figure 1:**
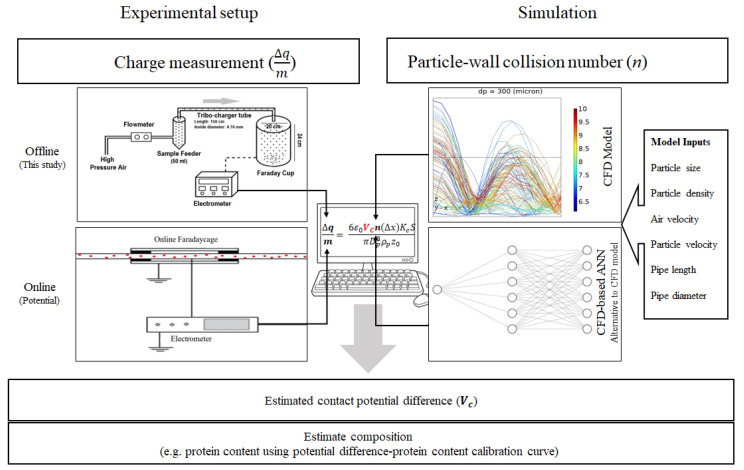
Schematic illustration of experimental (**Left**), and computational section (**Right**) of powders characterization method based on experimental charge measurement and computational estimation of particle-wall collision numbers.

The experimental setup for powder tribocharging and charge measurements consists of three sections, including a sample feeder, a tribocharging tube, and a Faraday cup connected to an electrometer, as illustrated in Figure 1. The sample feeder is a 50 mL container that is connected to a high-pressurized air supply, and the airflow rate is adjusted using a flowmeter before introducing dry air at the bottom of the sample feeder. The tribocharging unit is a polytetrafluoroethylene (PTFE) tube with 150 cm length and 4.76 mm inner diameter. At the inlet, the tribocharger tube is connected to the sample feeder, and at the outlet, to a Faraday cup (Monroe Electronics Inc., Lyndonville, NY, USA) that is connected to an electrometer (Model 6514, Keithley Company, Cleveland, OH, USA) for charge measurements. 

Sieved yellow pea powders at various particle sizes (25 and 380 microns) and protein contents were used as a model system for the charge measurement experiments. Twenty grams of samples were initially placed inside the sample feeder, and dry air was introduced at the bottom of the sample feeder at the adjusted airflow rate of 7 LPM. The solid mass flow rate was 1 (g/min). Particles were fluidized in the feeder, and only a few of them entered the charging tube at a time, which satisfied the dilute condition. During transfer within the charging tube, particles repeatedly collide with the wall, and the charge transfer occurs due to different work functions of contact surfaces. Charged particles are directly dispensed into the Faraday cup, where an electrometer measures the potential difference that occurs between the inner wall and outer wall of the Faraday cup. The mass of the particles accumulated in the Faraday cup was measured with an analytical balance, and the measurements were reported as the charge per mass ratio of the samples. All charge measurements were performed with at least ten replicates at room temperature for each particle size group, and the entire apparatus was flushed with dry air between each measurement to avoid any inaccuracy through tribocharging analysis.

### 3.5. Computational Fluid Dynamics and Artificial Neural Networks

Computational fluid dynamics (CFD) was used for the numerical simulation of particle trajectories, and computation of particle–wall collision numbers in particle-laden pipe flows using COMSOL Multiphysics^®^ (version 5.5). Reynolds averaged Navier–Stokes (RANS) equations were used for modeling the particle-laden flow, and the standard k-ε formulation was used for turbulence modeling. To solve the RANS equations, the Eulerian framework was used for the gas phase, and the solid phase was solved using the Lagrangian framework. The particle-laden flow was assumed to be in the dilute phase, which means that the volume fraction of the solid phase is low compared to the volume fraction of the fluid phase. There is no parameter that clearly establishes a threshold between dilute and dense flows. As per the well-known Elghobashi’s map, particle volume fraction threshold values define the boundaries between the different flow regimes. One-way coupling occurs for solid volume fractions lower than 10^−6^ [32]. Investigation of the particle–wall interactions in a dilute particle-laden flow is of interest in this study. Therefore, particle-particle interactions are neglected, and solid and gas phases are coupled in a one-way manner. The drag force and gravity are assumed to be the dominant factors in determining the particle trajectories. The Schiller–Naumann model was used for describing the drag coefficient. This model is suitable for dilute flows with rigid spherical particles [33]. The drag coefficient in this model is defined as

(5) $$C_{d}=\{\begin{array}{ll} \frac{24}{Re_{p}}\left(1+0.15Re_{p}^{0.687}\right) & Re_{p\;}<1000\\ 0.44 & Re_{p\;}>1000 \end{array}$$

Particle transport in particle-laden flows is affected by various parameters related to the solid phase properties (particle size, density, and shape) or the conveying line properties (gas velocity, gas pressure, and pipe geometry). Accounting for these parameters and properties, a novel method to predict particle–wall collision numbers in particle-laden flows is to use artificial neural networks (ANN). The latest advances in artificial neural networks have paved the way to develop a tool for analyzing non-linear systems, such as particle-laden flows. ANNs are competent in learning the complicated correlations between various variables by assigning weights and biases attached to the neurons [34]. Determining the appropriate network topology for a specific problem is an influential step that affects the network performance and liability. Therefore, an effective network architecture requires high expertise in identifying proper hyperparameters for the network, such as the number of hidden layers, the number of neurons in each layer, and a knowledgeable background in the corresponding field [35]. Hence, this topic has been the subject of enormous research in recent years [36,37]. The computation of particle–wall collision numbers for different combinations of input parameters through CFD analysis is extremely time-intensive, taking hours if not days to run every simulation. An ANN which was capable of learning complex relationships between input variables and output values was used to overcome this obstacle by reducing the computation time to a few seconds. The ANN model presented in this research was trained based on the various physical parameters of particles and flow systems and the collision numbers simulated through the computationally-intensive CFD model. An ANN with six inputs, namely particle size, particle density, air velocity, the vertical velocity of particles, pipe diameter, and pipe length, was designed and trained to predict particle–wall mean collision numbers. A brief summary of the database generated for developing a neural network, along with the inputs and output range, is presented in Table 1. 

**Figure 2:**
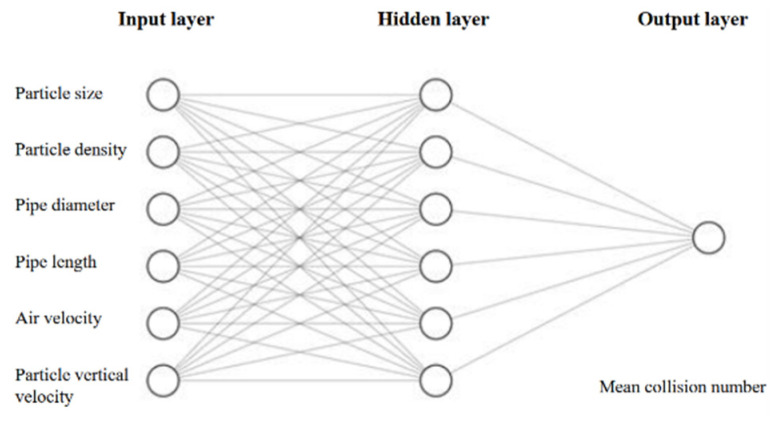
The architecture of the neural network used for fast computation of collision numbers using six input parameters and a hidden layer with six neurons.

The values of the input variables are highly scattered by several orders of magnitude, which makes it challenging to explore the influence of each parameter on particle–wall mean collision numbers, labeled as the model output. Therefore, data normalization is a vital step in preparing data for artificial neural networks. For this purpose, Min-Max normalization was used, and all data points were normalized between 0 and 1. A three-layer neural network was used to train the prepared database (Figure 2). Hence, 70% of the database, including 137 data points, was assigned for the training set, 15% of the database (30 data points) was employed for the validation of the network, and the remaining 15%, including 30 data points, was employed for testing the network performance and is called ‘test data’. The network was not trained with the data in the test set. To obtain a realistic network performance, 30 neural networks with different initial weights and biases were generated using a similar training set, and the average value of RMSEs calculated for all 30 networks was considered to evaluate the network performance.

## 4. Results and Discussion

### 4.1. Experimental Results (Characterization of All Sieved Fractions in Terms of Partice Size, Protein Content and Charge-to-Mass Ratio)

**Figure 3:**
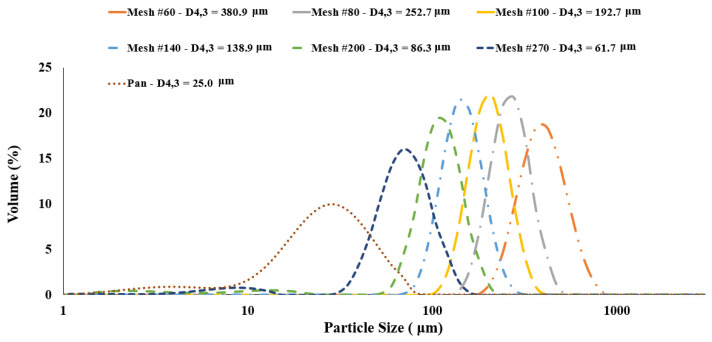
Partticle size distributions of yellow pea sieved fractions.

All sieved fractions were analyzed for particle size, protein content and charge-to-mass (nC/g) ratios to be coupled with the tribo-charging and CFD models, to estimate the contact potential difference of powders. Particle size distribution curves of all yellow pea sieved fractions are shown in Figure 3. Fractions sieved using mesh numbers 60, 80, 100, 140 and 200 yielded monomodal (narrow peaks) size distribitions, while the fraction sieved at mesh number 270 along with the pan fraction represented bimodal distributions with one shallow peak less than 10 μm. The pan fraction exhibited a very wide distribution of small size paticles. As expected, the fractions’ mean particle sizes (D4,3) reduced significantly from 380.9 to 61.7 μm by increasing the mesh numbers. The pan fraction had the smallest particle size of 25 μm.

Table 2 represents the protein contents of all fractions in relation to their mean particle size and charge-to-mass ratios (nC/g). Other than the pan fraction (<53 μm) having a wide particle size distribution, the general trend would be that the sieved fractions with larger mean diameters contain lower protein contents, thus acquiring less charge-to-mass (nC/g) ratios. Table 2 shows the charge-to-mass ratio of particles with different diameters. The lowest charge-to-mass ratio gained via tribocharging belonged to the small particles with 25-micron (micrometer) diameter, whereas particles with approximately 60-micron (61.7 microns to be exact) diameter showed an extremely high charge-to-mass ratio after tribocharging. At the same time, the measured charge-to-mass ratio for 60-micron particles showed the highest standard deviation between different experimental runs. For particles larger than 60-microns, the amount of charge-to-mass gained by the particles during tribocharging showed a sharp decline for 86-micron particles and gradually decreased for particles larger than 200-micron.

### 4.2. Influence of Particle Size on Particle-Wall Collision Numbers

**Figure 4:**
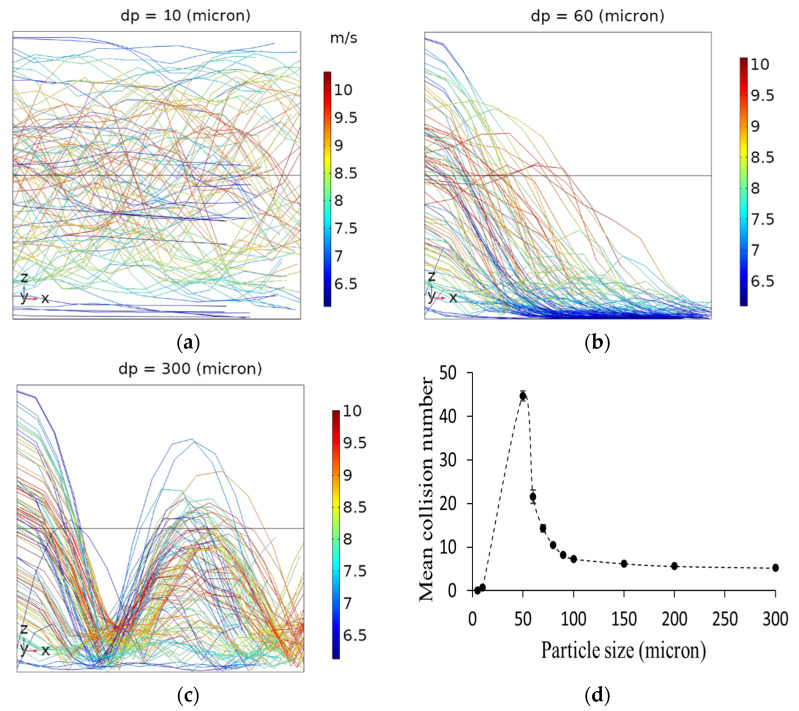
CFD analysis of particle trajectories for (**a**) low; (**b**) intermediate; (**c**) high Stk; and (**d**) influence of particle size on calculated particle-wall mean collision numbers. Particles were released into a 1 m pipe with a 4.76 mm inner diameter, and the air velocity was 8.4 (m/s). The color of trajectory lines shows the velocity magnitude (m/s) of particles. The *x*-axis points in the streamwise direction, the *z*-axis in the wall-normal direction, and the *y*-axis in the spanwise direction.

To investigate the influence of particle size on the particle–wall interactions, particles with the same densities but different diameters ranging from 5 to 300 microns were released into a pipe with a length of 1 m and diameter of 4.76 mm. The air velocity in the pipe was 8.4 m/s, and 100 particles of each size were released into the system in every simulation. The average collision number was expressed as the mean collision number. The CFD results confirmed that the mean collision number slightly changed when releasing 100 particles into the system compared to 10 released particles due to the random release of particles at the entrance. While a further increase in the number of released particles (i.e., 500, 1000, 2000, and 5000) substantially increased the computation time, no significant difference was observed for the calculated mean collision numbers. Therefore, releasing 100 particles was found to be a realistic number with a reasonable computation time. As a result, the release of 100 particles was considered as the base number for all other simulations. Particles were categorized into three different groups based on the Stokes numbers, namely, low Stokes (Stk < 1), moderate Stokes (1 < Stk < 10), and high Stokes particles (Stk > 10). The particle Stokes number (Stk) was defined as the ratio between the particle response time ($\tau_{p}$), and the characteristic timescale of the fluid flow ($\tau_{L}$). The particle response time (particle relaxation time) was the time required by the particle to respond to eddies in the flow. The value of particle response time depends on the particle size (*d_p_*), particle density ($\rho_{p}$), and fluid viscosity (μ). Assuming that the turbulent timescale is constant, particles with a larger diameter and higher density require a longer time to respond to the fluid structures than particles with lower densities and smaller sizes. These results were in good agreement with those obtained by Chan et al. conducting a direct numerical simulation of two-phase turbulent pipe flow to investigate the mechanism of particle transport within a range of Stk [38]. At a low Stk (i.e., 10-micron particles and smaller), particles act like tracers that follow the fluid streamlines, but they do not have enough inertia to cross the eddies. The turbulent structures govern the transportation of these particles entirely. Therefore, they barely collide with the wall (Figure 4a). At intermediate Stk (e.g., 60-micron particles), particles have enough inertia (e.g., due to gravity) to cross the eddies but not enough inertia to get back to the center streamlines. Therefore, they are trapped in low-velocity zones near the wall and repeatedly collide with the wall until they leave the pipe (Figure 4b). The transport mechanism of particles with intermediate Stk is affected by the turbulent structures and particle inertia. At high Stk (e.g., 300-micron particles), the effect of turbulent structures on particle transport is less pronounced than the intermediate Stk values because these particles have high inertia, enabling them to cross the eddies and bounce back into the bulk medium after colliding with the wall (Figure 4c). Generally, the trajectory of particles with large Stk is unresponsive to the short timescales of the turbulent flow. Figure 4d shows the calculated mean collision numbers for different particle sizes released into a 1 m pipe with 4.76 mm inner diameter and 8.4 (m/s) average air velocity.

### 4.3. Influence of Particle Density on Particle-Wall Collision Numbers

**Figure 5:**
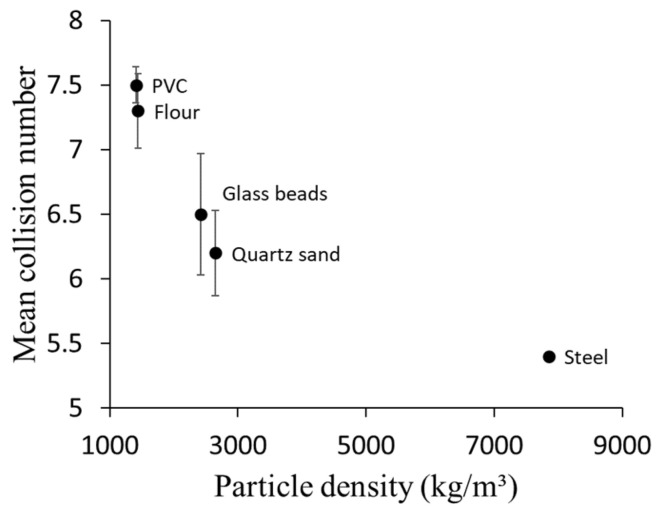
Comparing mean collision numbers of 100-micron particles with different densities. All particles assumed spherical particles released into a 1 m pipe with 4.76 mm inner diameter, and the air velocity was 8.4 (m/s).

Five different materials with different densities were investigated to understand the influence of particle density on particle–wall mean collision numbers. Table 3 shows the density of particles used in simulations. In each density group, the mean collision numbers were calculated for 100-micron particles. Figure 5 shows that for different densities, the particle–wall collision number decreases by increasing the particle density. This could be explained by the direct relationship between particle density and particle response time. As particle density increased, much more time was required to respond to the changes in the turbulent flow, as was expected for particles with a high Stk. Therefore, particles with higher densities do not have enough time to follow turbulent streamlines, and their motion is governed mainly by their inertia.

### 4.4. Influence of Pipe Diameter and Length on Particle-Wall Collision Numbers

**Figure 6:**
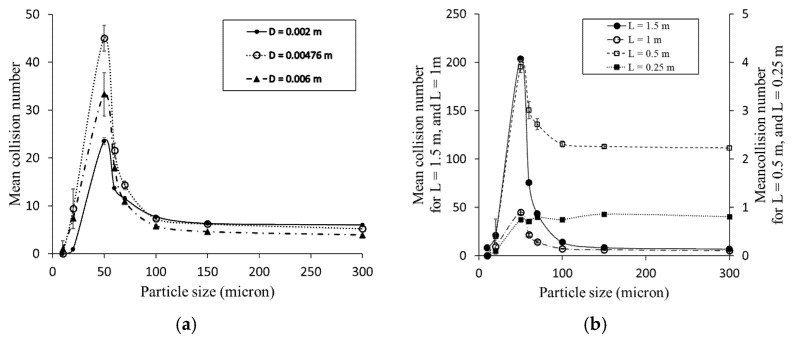
(**a**) Influence of pipe diameter on particle-wall mean collision number. In all cases, particles were released into a 1 m pipe with 8.4 (m/s) air velocity; (**b**) influence of pipe length on particle-wall interactions. In all cases, particles were released into a pipe with 4.76 mm inner diameter and 8.4 (m/s) air velocity.

Several CFD simulations were performed using different pipe diameters and lengths, with an average air velocity of 8.4 m/s, to investigate the influence of these parameters on the particle–wall mean collision numbers. Figure 6a shows that for particles larger than 100 microns (high Stk particles), the mean collision numbers decrease by pipe diameter since the distance between particles and the walls increases, and consequently, particles have to travel a longer distance to reach the wall. For particles smaller than 100-micron, the mean collision number was higher in 4.76 mm inner diameter pipes than in 2 mm pipes mostly because in the larger pipe diameter, the integral timescale of turbulence increased and turbulent dispersion was enhanced for smaller particles, resulting in more collisions with the wall. Similar results were confirmed by Sommerfeld [39].

Four different pipe lengths (0.25, 0.5, 1, and 1.5 m) were used in simulations to investigate the influence of pipe length on the particle–wall mean collision numbers. In all simulations, the pipes’ diameter and airflow velocity were 4.76 mm and 8.4 m/s, respectively. For particles with different sizes released in the pipe of 0.25-m length, the calculated mean collision numbers were almost similar, i.e., less than one collision per particle, which means that in such conditions, nearly all particles exit the pipe without colliding with the pipe wall. By increasing the pipe length to 0.5-m, different particle sizes showed different mean collision numbers, but there is still no significant difference between various particle sizes in terms of the calculated mean collision number. For particles released into a 1 m length pipe, there is a clear difference in recorded mean collision numbers for different particle sizes. Based on these results, the minimum pipe length for investigating particle size’s effect on mean collision numbers is 1 m pipe length because, in shorter pipes, all particle sizes show almost similar interactions with the wall. By increasing the pipe length to 1.5 m, the mean collision numbers for particles larger than 100 microns increased as the particle’s residence time increased. For particles smaller than 100 microns, as particle motion was governed mostly by the turbulent structures, the slope of this augmentation was high (Figure 6b).

### 4.5. Influence of Air Velocity on Particle-Wall Collision Numbers

**Figure 7:**
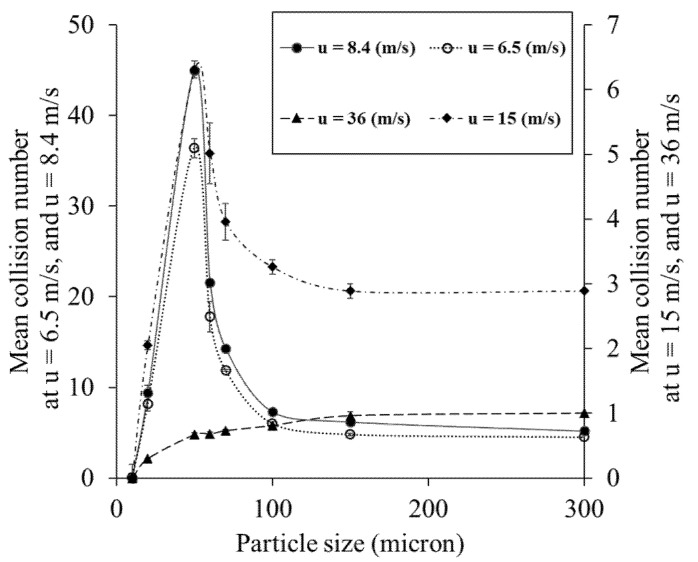
Influence of air velocity on particle-wall mean collision numbers. All calculations were accomplished for a single pipe length (1 m) and diameter (4.76 mm).

To study the impact of air velocity on particle–wall interactions, particles of different sizes were released into pipe flows with different air velocities. The calculated mean collision numbers were also compared in a laminar and turbulent flow. The pipe dimension used for simulations was 1 m in length and 4.76 mm in diameter in all cases. One hundred particles were released in every simulation, and the average collision number was expressed as the mean collision number. Simulation results confirmed that particle–wall mean collision number and air velocity are inversely related (Figure 7). Comparing particles with similar size traveling in different air velocities confirmed that more particle–wall interaction occurs in lower air velocity (i.e., higher particle residence time), whereas in higher air velocity (i.e., lower particle residence time), particles barely collide with the pipe wall before exiting the pipe. Results also show that at lower air velocities, particle–wall mean collision numbers decrease with particle size. Interestingly, in very high air velocities (36 m/s), large particles showed more collisions than smaller sizes. This is most likely due to smaller particles passing through high air velocities being trapped at the center eddies of the pipe flow and exiting the pipe before the gravitational force pulls them towards the pipe wall.

Furthermore, to investigate the effect of turbulence on particle–wall interactions, a laminar flow with 6.5 m/s air velocity was compared with turbulent flows in higher velocities. Figure 7 shows that the particle–wall mean collision number in laminar flow is higher than that in the turbulent flows with 15 and 36 m/s due to higher particle residence time. On the other hand, it shows fewer interactions than turbulent flow when the air velocity is 8.4 m/s.

### 4.6. Neural Network Performance

**Figure 8:**
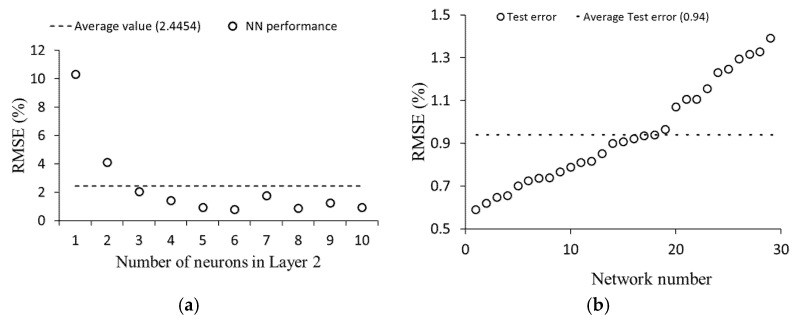
(**a**) influence of number neurons in the second layer on network performance; (**b**) the average performance of the neural network.

A three layer ANN was used with one input layer, one output layer, and one hidden layer. The input and output layers had six neurons and one neuron, respectively, corresponding to the input parameters (particle size and density, pipe length and diameter, particle vertical velocity, and air velocity) and output parameter (particle–wall mean collision number). The hidden layer is an intermediate layer between the input and the output layer, and an activation function influences every neuron in this layer. Deciding the proper number of hidden layers and the number of neurons in each hidden layer is essential for the neural network’s performance [40] and many studies have been devoted to the evaluation of the number of neurons in the hidden layer. Determining the size of the hidden layer neurons is often a challenging stage as it is influenced by different parameters such as the activation function, the algorithm used for training, and the training data that the model is trained with. In some applications where a long training time is not critical, and the model’s accuracy is the first concern, multiple hidden layers are used. However, using multiple hidden layers would result in overfitting of the model [41]. Developers have been using different techniques for optimizing the number of neurons in the hidden layer, such as using least squares estimation [42], pruning the hidden nodes [43], or using the two phase method [44]. To determine the optimal number of neurons in the hidden layer (Layer 2), an optimization analysis was performed in MATLAB^®^. In this analysis, the number of neurons in the hidden layer (Layer 2) was altered from 1 to 10. The root mean square error (RMSE) of each network was calculated five times, and the average value was considered as the performance of the generated networks. Figure 8a summarizes the RMSEs of different networks as a function of the number of neurons in the hidden layer. It can be concluded from the graph that using six neurons in Layer 2 could obtain a high accuracy from the model.

The test set, containing 15% of the database, was employed to evaluate the neural network performance. This set includes new data for the model, and the model did not use it during the training process. In assessing the model performance, 30 neural networks with various initial weights and bias values were generated using the same training data set. The neural network prediction values of the test set were expressed as the average output values of all 30 networks. Figure 8b summarizes the error from the 30 networks when applied to the test data set. The average value of RMSE for all networks was low (0.94), which demonstrates the model’s excellent performance in predicting the mean collision numbers for the test data set.

### 4.7. Correlation of Experimentally Measured Charge with Calculated Collision Numbers

**Figure 9:**
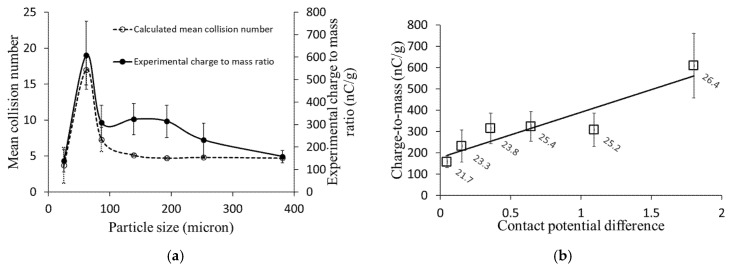
(**a**) Effect of particle size on mean collision number and charge-to-mass ratio; (**b**) correlation of experimentally measured charge-to-mass ratios with calculated contact potential difference for sieved samples with different particle sizes and different protein content percentages (data labels). Error bars show the deviation of charge measurements during multiple experimental runs.

Figure 9a describes the influence of particle size on the particle–wall mean collision numbers and the experimentally measured charge-to-mass ratios. The lowest charge-to-mass ratio was reported for small particles with a 25-micron diameter, having the minimum mean collision number (3.7 collisions per particle) in this graph. On the other hand, the maximum charge-to-mass ratio was reported for 60-micron particles that have the highest particle–wall mean collision number (17 collisions per particle). For larger particles, the measured charge-to-mass ratio decreases exponentially due to fewer particle–wall interactions, and for particles larger than 100 microns, the charge-to-mass ratios do not change dramatically as the collision numbers are almost identical. The correlation between the experimental charge measurement values and the computed particle–wall mean collision numbers for different particle sizes is shown in Figure 9a. These results confirm the correlation between particles’ charging rate and the number of particle–wall interactions. Hence, the particle–wall collision number computed based on different particle and flow properties is an essential parameter for modeling particle tribocharging in particle-laden flows.

### 4.8. Influence of Powder Composition on Charging Behavior

The material of the contact bodies significantly affects the tribocharging process [25,45,46]. This fact has been applied as a tool for tribo-separation of macroparticles in the recycling industry [47,48,49,50] and for the tribo-separation of microparticles, such as protein and starch particles on a lab scale [14,20,51]. The contact potential difference between the contact surfaces depends on the physicochemical properties of the contact material and requires costly laboratory equipment, and a controlled environment to measure. In this study, the contact potential difference was calculated from the experimental charge measurement data, and the CFD and tribocharging models. The results from this study also revealed a strong correlation between the calculated contact potential difference and the protein content of powder samples.

In this study, numerical modeling of particle tribocharging was conducted based on the particle–wall collision numbers calculated via CFD at different operating conditions. The contact potential difference (CPD), which is a function of contact surfaces’ work functions and material, was calculated from the experimental charge measurement results. Equation (2) was used to calculate the contact potential difference (*V_c_*) for samples with a known particle size using the predicted particle–wall collision numbers (estimated via CFD or ANN model) and the experimental tribocharge data. Figure 9b shows the correlation between the measured charge-to-mass ratios and the calculated CPD values for samples with different protein contents. Results also confirmed that the value of contact potential difference increases with the protein content of the samples. It is clear from the graph that the charge-to-mass ratio of particles is strongly correlated to the contact potential difference, explaining the high charging tendency of powders with higher protein contents. This means that the charge measurement during powder transport in particle-laden flows could be used as a potential tool to characterize powders’ physical and chemical properties (e.g., particle size or protein content).

## 5. Limitations

Powder tribocharging during the handling process and transportation in a gas-solids pipe flow is influenced by numerous parameters and accounting for all of them in a single charge transfer model is currently unrealistic. The main focus of the CFD model in this study was to investigate the influence of the number of particle–wall collisions, which is affected by the characteristics of particles and flow, on the tribocharging behavior of powders. However, future works are required to modify the proposed CFD and tribocharging model by measuring the contribution of humidity, particle sphericity, surface geometry, geometry of the tribocharger, surface roughness of contact surfaces, dust and impurities, and different solid loadings to the tribocharging behavior of powders. Furthermore, the experimental results provided in this study are preliminary, and future experimental studies are required to further investigate the influence of chemical composition on powder tribocharging. Although the findings are preliminary, the proposed method could be a benchmark that could be used as a potential approach for process monitoring in various manufacturing processes for the quality control of final products.

## 6. Conclusions

In summary, developing a rapid, cost-effective, and non-destructive method for the online monitoring of powder concentrations is sought after in the pharmaceutical industry, food fraud assessment, and quality control of particulate products. This research investigated the charging behavior of powders during tribocharging in particle-laden flows as a potential tool for identifying the chemical composition and concentration of powders’ constituents. The numerical model developed for tribocharging of particles in particle-laden flows is suitable for simulating the charge generation experienced by different particle sizes of similar material. Furthermore, a strong correlation was observed between the values of calculated contact potential difference for samples with different protein contents and the corresponding measured charge-to-mass ratios. Hence, the online characterization of powders, such as particle size determination and concentration of various constituents (e.g., protein content), during transport in particle-laden pipe flows could be a novel application of the proposed numerical model. Future investigations could be focused on understanding the tribocharging behavior of powders with different materials and the influence of different chemical concentrations on powder charging during transport in pipes of different materials.

Overall, tribocharging is a fairly unknown phenomenon, and a better understanding of it not only helps to prevent the drawbacks, such as the risk of fire explosion and wall-fouling, but could also help to improve current powder handling processes and develop new applications such as powder characterization and online monitoring of powder constituents. Powder tribocharging during transport in particle-laden flows is strongly affected by the physical and chemical properties of powders. Although powder tribocharging during transport is often considered a nuisance phenomenon resulting in dust explosion, it could be utilized as an accessible and powerful tool for manipulating powders’ characteristics if well understood and controlled. It is time to think of this mysterious natural phenomenon not only as a negative issue that should be avoided, but as an endless source of energy that could be utilized in various applications.

## Figures and Tables

**Table 1:** Network inputs and output.

Parameter	Range
**Network inputs**	
Particle size (micron)	10–600
Particle density (kg/m^3^)	1410–7850
Pipe diameter (m)	0.002–0.006
Pipe length (m)	0.25–1.5
Average air velocity (m/s)	6.5–36
Particle vertical velocity (m/s)	0–1.2
**Network output**	
Particle-wall mean collision number	0.6–80

**Table 2:** Mean diameter (D4,3), protein content and charge-to-mass ratio (nC/g) of yellow pea sieved fractions.

Sieved Fractions (Mesh Numbers)	Mean Particle Diameter (D43, µm)	Protein Content (wt%)	Charge-to-Mass Ratio (nC/g)
No. 60	380.9 ± 9.9	21.7 ± 0.9	157.9 ± 27.8
No. 80	252.7 ± 1.5	23.3 ± 0.2	231.2 ± 74.9
No. 100	192.7 ± 0.5	23.8 ± 0.7	314.5 ± 71.6
No. 140	138.9 ± 0.1	25.4 ± 0.7	323.8 ± 69.1
No. 200	86.3 ± 0.1	25.2 ± 0.4	307.8 ± 78.3
No. 270	61.7 ± 0.0	26.4 ± 0.8	608.8 ± 151.3
Pan (<53 μm)	25.0 ± 0.0	14.2 ± 0.1	138.2 ± 49.5

**Table 3:** Density of materials used in simulations.

Material	Density (kg/m^3^)
Flour	1440
PVC	1410
Glass beads	2420
Quartz sand	2650
Steel	7850

## References

[1] Matsusaka S., Maruyama H., Matsuyama T., Ghadiri M. (2010). Triboelectric charging of powders: A review. Chem. Eng. Sci..

[2] Lim T.S.E., Lim E., Tay W.Q., Cruz D.A., Wong S.Y. (2021). Triboelectric charging of 3-in-1 coffee mixes: Formulation and fouling. J. Food Process Eng..

[3] Pu Y., Mazumder M., Cooney C. (2009). Effects of Electrostatic Charging on Pharmaceutical Powder Blending Homogeneity. J. Pharm. Sci..

[4] Hi M.A., Nourafkan E., Hassanpour A. (2018). A review of current techniques for the evaluation of powder mixing. Adv. Powder Technol..

[5] Hao T., Tukianen J., Nivorozhkin A., Landrau N. (2013). Probing pharmaceutical powder blending uniformity with electrostatic charge measurements. Powder Technol..

[6] Schuck P., Dolivet A., Jeantet R. (2012). Analytical Methods for Food and Dairy Powders.

[7] Vitelli M., Mehrtash H., Assatory A., Tabtabaei S., Legge R.L., Rajabzadeh A.R. (2021). Rapid and non-destructive determination of protein and starch content in agricultural powders using near-infrared and fluorescence spectroscopy, and data fusion. Powder Technol..

[8] Yu L.X. (2008). Pharmaceutical Quality by Design: Product and Process Development, Understanding, and Control. Pharm. Res..

[9] Khan A., Munir M.T., Yu W., Young B.R. (2020). A Review Towards Hyperspectral Imaging for Real-Time Quality Control of Food Products with an Illustrative Case Study of Milk Powder Production. Food Bioprocess Technol..

[10] Fotovat F., Bi X.T., Grace J.R. (2017). Electrostatics in gas-solid fluidized beds: A review. Chem. Eng. Sci..

[11] Amyotte P. (2013). An Introduction to Dust Explosions: Understanding the Myths and Realities of Dust Explosions for a Safer Workplace.

[12] Chowdhury F., Sowinski A., Ray M., Passalacqua A., Mehrani P. (2018). Charge generation and saturation on polymer particles due to single and repeated particle-metal contacts. J. Electrost..

[13] Assatory A., Vitelli M., Rajabzadeh A.R., Legge R.L. (2019). Dry fractionation methods for plant protein, starch and fiber enrichment: A review. Trends Food Sci. Technol..

[14] Tabtabaei S., Jafari M., Rajabzadeh A.R., Legge R.L. (2016). Solvent-free production of protein-enriched fractions from navy bean flour using a triboelectrification-based approach. J. Food Eng..

[15] Tabtabaei S., Jafari M., Rajabzadeh A.R., Legge R.L. (2016). Development and optimization of a triboelectrification bioseparation process for dry fractionation of legume flours. Sep. Purif. Technol..

[16] Tabtabaei S., Vitelli M., Rajabzadeh A.R., Legge R. (2017). Analysis of protein enrichment during single- and multi-stage tribo-electrostatic bioseparation processes for dry fractionation of legume flour. Sep. Purif. Technol..

[17] Tabtabaei S., Konakbayeva D., Rajabzadeh A.R., Legge R.L. (2019). Functional properties of navy bean (Phaseolus vulgaris) protein concentrates obtained by pneumatic tribo-electrostatic separation. Food Chem..

[18] Jafari M., Rajabzadeh A.R., Tabtabaei S., Marsolais F., Legge R.L. (2016). Physicochemical characterization of a navy bean (Phaseolus vulgaris) protein fraction produced using a solvent-free method. Food Chem..

[19] Basset C., Kedidi S., Barakat A. (2016). Chemical- and Solvent-Free Mechanophysical Fractionation of Biomass Induced by Tribo-Electrostatic Charging: Separation of Proteins and Lignin. ACS Sustain. Chem. Eng..

[20] Wang J., Zhao J., de Wit M., Boom R.M., Schutyser M.A. (2016). Lupine protein enrichment by milling and electrostatic separation. Innov. Food Sci. Emerg. Technol..

[21] Laurentie J., Traoré P., Dascalescu L. (2013). Discrete element modeling of triboelectric charging of insulating materials in vibrated granular beds. J. Electrost..

[22] Dwari R.K., Rao K.H. (2007). Dry beneficiation of coal—A review. Miner. Process. Extr. Met. Rev..

[23] Crowe C.T., Schwarzkopf J.D., Sommerfeld M., Tsuji Y. (2011). Multiphase Flows with Droplets and Particles.

[24] Masuda H., Matsusaka S., Akiba S., Shimomura H. (1998). Electrification of Fine Particles in Gas-Solids Pipe Flow. KONA Powder Part. J..

[25] Itakura T., Masuda H., Ohtsuka C., Matsusaka S. (1996). The contact potential difference of powder and the tribo-charge. J. Electrost..

[26] Masuda H., Komatsu T., Iinoya K. (1976). The static electrification of particles in gas-solids pipe flow. AIChE J..

[27] Matsusaka S., Ghadiri M., Masuda H. (2000). Electrification of an elastic sphere by repeated impacts on a metal plate. J. Phys. D Appl. Phys..

[28] Watanabe H., Ghadiri M., Matsuyama T., Ding Y.L., Pitt K.G., Maruyama H., Matsusaka S., Masuda H. (2007). Triboelectrification of pharmaceutical powders by particle impact. Int. J. Pharm..

[29] Ema A., Yasuda D., Tanoue K.-I., Masuda H. (2003). Tribo-charge and rebound characteristics of particles impact on inclined or rotating metal target. Powder Technol..

[30] Tanoue K.-I., Tanaka H., Kitano H., Masuda H. (2001). Numerical simulation of tribo-electrification of particles in a gas–solids two-phase flow. Powder Technol..

[31] Matsusaka S., Masuda H. (2003). Electrostatics of particles. Adv. Powder Technol..

[32] Elghobashi S. (1991). Particle-laden turbulent flows: Direct simulation and closure models. Appl. Sci. Res..

[33] Schiller L., Naumann Z. (1935). A Drag Coefficient Correlation. Z. Ver. Deutsch. Ing..

[34] Swiniarski R. (1996). Introduction to Neural Networks. Neural Networks World.

[35] Anochi J.A., Velho H.F.D.C. Optimization of Feedforward Neural Network by Multiple Particle Collision Algorithm. Proceedings of the IEEE SSCI 2014–2014 IEEE Symposium Series on Computational Intelligence—FOCI 2014: 2014 IEEE Symposium on Foundations of Computational Intelligence.

[36] Ramchoun H., Idrissi M.A.J., Ghanou Y., Ettaouil M. (2016). Multilayer Perceptron: Architecture Optimization and Training. Int. J. Interact. Multimed. Artif. Intell..

[37] Rajabzadeh A.R., Zendehboudi S., Lohi A., Elkamel A. (2013). Colloidal interaction and connectionist modelling of protein osmotic pressure and the effect of physicochemical properties. Can. J. Chem. Eng..

[38] Chan L., Zahtila T., Ooi A., Philip J. (2021). Transport of particles in a turbulent rough-wall pipe flow. J. Fluid Mech..

[39] Sommerfeld M. (2003). Analysis of collision effects for turbulent gas–particle flow in a horizontal channel: Part I. Particle transport. Int. J. Multiph. Flow.

[40] Karsoliya S. (2012). Approximating Number of Hidden layer neurons in Multiple Hidden Layer BPNN Architecture. Int. J. Eng. Trends Technol..

[41] Liu Y., Starzyk J.A., Zhu Z. Optimizing Number of Hidden Neurons in Neural Networks. Proceedings of the IASTED International Conference on Artificial Intelligence and Applications, AIA.

[42] Rivals I., Personnaz L. A Statistical Procedure for Determining the Optimal Number of Hidden Neurons of a Neural Model. Proceedings of the Second International Symposium on Neural Computation (NC).

[43] Fnaiech F., Najim M. A New Feedforward Neural Network Hidden Layer Neuron Pruning Algorithm. Proceedings of the ICASSP, IEEE International Conference on Acoustics, Speech and Signal.

[44] Shin-Ike K. A Two Phase Method for Determining the Number of Neurons in the Hidden Layer of a 3-Layer Neural Network. Proceedings of the SICE Annual Conference.

[45] Biegaj K.W., Rowland M.G., Lukas T.M., Heng J.Y.Y. (2017). Surface Chemistry and Humidity in Powder Electrostatics: A Comparative Study between Tribocharging and Corona Discharge. ACS Omega.

[46] Mazumder M., Sims R., Biris A., Srirama P., Saini D., Yurteri C., Trigwell S., De S., Sharma R. (2006). Twenty-first century research needs in electrostatic processes applied to industry and medicine. Chem. Eng. Sci..

[47] Dodbiba G., Fujita T. (2004). Progress in Separating Plastic Materials for Recycling. Phys. Sep. Sci. Eng..

[48] Pearse M., Hickey T. (1978). The separation of mixed plastics using a dry, triboelectric technique. Resour. Recover. Conserv..

[49] Żenkiewicz M., Żuk T., Markiewicz E. (2015). Triboelectric series and electrostatic separation of some biopolymers. Polym. Test..

[50] Yang J., Wang H., Zhang G., Bai X., Zhao X., He Y. (2019). Recycling organics from non-metallic fraction of waste printed circuit boards by a novel conical surface triboelectric separator. Resour. Conserv. Recycl..

[51] Landauer J., Foerst P. (2018). Triboelectric separation of a starch-protein mixture—Impact of electric field strength and flow rate. Adv. Powder Technol..

